# A Targeted Computational Screen of the SWEETLEAD Database Reveals FDA-Approved Compounds with Anti-Dengue Viral Activity

**DOI:** 10.1128/mBio.02839-20

**Published:** 2020-11-10

**Authors:** Jasmine Moshiri, David A. Constant, Bowen Liu, Roberto Mateo, Steven Kearnes, Paul Novick, Ritika Prasad, Claude Nagamine, Vijay Pande, Karla Kirkegaard

**Affiliations:** a Department of Microbiology and Immunology, Stanford University School of Medicine, Stanford, California, USA; b Department of Genetics, Stanford University School of Medicine, Stanford, California, USA; c Department of Biology, Stanford University, Stanford, California, USA; d Department of Chemistry, Stanford University, Stanford, California, USA; e Department of Comparative Medicine, Stanford University School of Medicine, Stanford, California, USA; St. Jude Children’s Research Hospital

**Keywords:** antiviral pharmacology, capsid, computational biology, dengue virus, proteases, repurposing

## Abstract

Affordable and effective antiviral therapies are needed worldwide, especially against agents such as dengue virus that are endemic in underserved regions. Many antiviral compounds have been studied in cultured cells but are unsuitable for clinical applications due to pharmacokinetic profiles, side effects, or inconsistent efficacy across dengue serotypes. Such tool compounds can, however, aid in identifying clinically useful treatments. Here, computational screening (Rapid Overlay of Chemical Structures) was used to identify entries in an *in silico* database of safe-in-human compounds (SWEETLEAD) that display high chemical similarities to known inhibitors of dengue virus. Inhibitors of the dengue proteinase NS2B/3, the dengue capsid, and the host autophagy pathway were used as query compounds. Three FDA-approved compounds that resemble the tool molecules structurally, cause little toxicity, and display strong antiviral activity in cultured cells were selected for further analysis. Pyrimethamine (50% inhibitory concentration [IC_50_] = 1.2 μM), like the dengue proteinase inhibitor ARDP0006 to which it shows structural similarity, inhibited intramolecular NS2B/3 cleavage. Lack of toxicity early in infection allowed testing in mice, in which pyrimethamine also reduced viral loads. Niclosamide (IC_50_ = 0.28 μM), like dengue core inhibitor ST-148, affected structural components of the virion and inhibited early processes during infection. Vandetanib (IC_50_ = 1.6 μM), like cellular autophagy inhibitor spautin-1, blocked viral exit from cells and could be shown to extend survival *in vivo*. Thus, three FDA-approved compounds with promising utility for repurposing to treat dengue virus infections and their potential mechanisms were identified using computational tools and minimal phenotypic screening.

## Abstract (precis)

No antiviral therapeutics are currently available for dengue virus infections. By computationally overlaying the three-dimensional (3D) chemical structures of compounds known to inhibit dengue virus over those of compounds known to be safe in humans, we identified three FDA-approved compounds that are attractive candidates for repurposing as antivirals. We identified targets for two previously identified antiviral compounds and revealed a previously unknown potential anti-dengue compound, vandetanib. This computational approach to analyze a highly curated library of structures has the benefits of speed and cost efficiency. It also leverages mechanistic work with query compounds used in biomedical research to provide strong hypotheses for the antiviral mechanisms of the safer hit compounds. This workflow to identify compounds with known safety profiles can be expanded to any biological activity for which a small-molecule query compound has been identified, potentially expediting the translation of basic research to clinical interventions.

## INTRODUCTION

Infectious disease poses a growing risk to global public health and disproportionately affects underprivileged populations. Dengue virus is responsible for an estimated 390 million infections each year (1), yet despite this enormous global public health and economic burden (2), there are currently no approved antivirals available to treat dengue infections. The costs of research and development and the specter of drug resistance are significant challenges associated with developing novel antiviral medications to combat this disease and others caused by RNA viruses (3). Dengue virus infects cells by receptor-mediated endocytosis of the enveloped virus. Within the endosome, acidification and ubiquitination (4) facilitate disassembly of the dengue viral capsid, an oligomer of dengue core protein, and the subsequent release of the single-stranded, positive-sense RNA genome. The viral RNA is translated in a continuous open reading frame to generate a large polyprotein that must be cleaved to free nonstructural and structural viral proteins. Dengue virus relies on host proteinases and its own viral proteinase, NS2B/3, to process this polyprotein. RNA replication is performed by negative-strand synthesis, followed by positive-strand synthesis, in membrane-associated complexes. Virion assembly also occurs in adjacent membranous complexes crafted from lipid droplets (5). Virions are subsequently processed through the Golgi for maturation and released through the secretory pathway while co-opting components of the cellular autophagy pathway for viral maturation and release (6). Throughout its infectious cycle, dengue virus relies on many other host factors to facilitate its growth as well (e.g., 7–14).

Developing effective antivirals is especially challenging because of the propensity for drug-resistant viral variants to emerge. Like most RNA viruses, dengue virus has a high mutation rate and exists as a quasispecies within every infected cell (15). This diversity can lead to the rapid emergence of drug resistance upon the application of selective pressure. Preferentially choosing compounds that suppress the rapid fixation of drug-resistant viral variants from the quasispecies can lower risk of antiviral resistance. One means by which this can be achieved is by combination therapy, in which multiple compounds are used so that the probability of the presence of a drug-resistant genome is reduced. Similarly, inhibiting the proviral activity of a host factor could reduce the probability of drug resistance because the mutation frequency of the host is low (16). Another approach is to accept that, for any individual compound, drug-resistant genomes will inevitably be formed during error-prone RNA replication. However, their outgrowth can be blunted if the remaining drug-susceptible genomes within the same cell are genetically dominant. Examples of such “dominant drug targets” are viral capsids, which are formed by the intracellular oligomerization of individual capsid proteins derived from multiple genomes (17, 18).

Other challenges associated with antiviral drug development may be alleviated by adopting strategic practices to reduce research and development expenses and timelines. The cost of bringing a single drug through the development pipeline, estimated at $2.6 billion U.S. dollars (USD), is extremely high and rising further (19). One strategy to bypass costly stages of the development pipeline and reduce time to market is to focus on repurposing compounds that have been previously approved for other indications (20). Through previous studies, these drugs will have undergone safety evaluations in preclinical models and patients, decreasing the risk of failure during phase I clinical trials or even allowing some clinical trial stages to be bypassed altogether. An additional strategy, the implementation of computational screening methods, can expedite and lower costs of preliminary high-throughput analyses compared to traditional first-round phenotypic screening.

**FIG 1:**
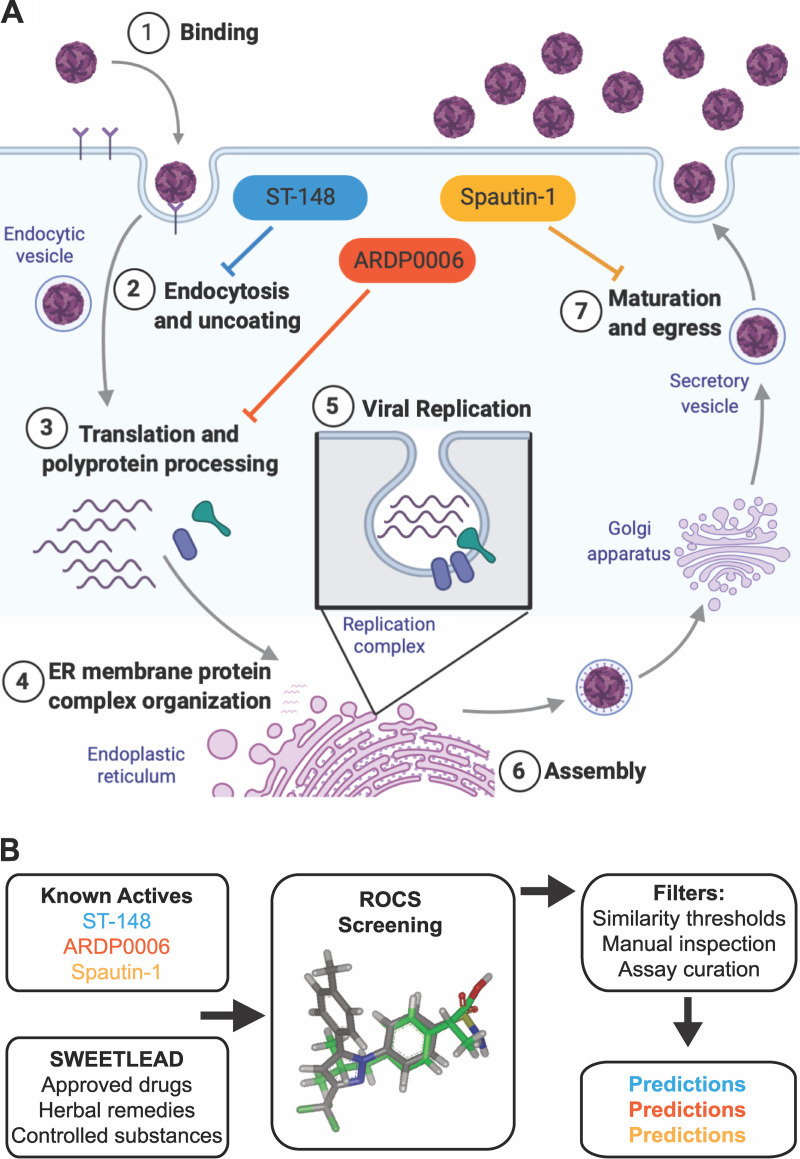
*In silico* screening to identify candidates for anti-dengue repurposing using known query compounds. (A) A single infectious cycle of dengue virus is shown diagrammatically to illustrate multiple steps at which antivirals can interfere. Core-binding molecules such as ST-148 could be directly virucidal, inhibit genome release (step 2), or interfere with assembly (step 6). Proteinase inhibitors such as ARDP0006 could derange polyprotein processing and could thus affect all subsequent stages that require mature proteins (step 3 and beyond). Inhibitors of autophagy and other membrane-sculpting processes could interfere with assembly of RNA replication complexes (step 5) or, like spautin-1, interfere with maturation and egress (step 7). The graphic was created with BioRender. (B) The SWEETLEAD *in silico* database of high-confidence chemical structures for FDA-approved compounds, medicinal herbs, and controlled substances was searched to identify those with high 3D shape and chemical similarity to query compounds ARDP0006, ST-148, and spautin-1. The Rapid Overlay of Chemical Structures (ROCS) virtual screening tool scores molecular similarity between pairs of molecules based on three-dimensional shape overlap and chemical similarity.

As is the case for most RNA viruses, treatment options for dengue infections are limited to supportive therapy and do not include direct-acting antivirals (21). However, many inhibitors of dengue virus with known targets have been reported in the literature. While these inhibitors have pharmacokinetic profiles, side effects, or inconsistent efficacy across dengue virus serotypes that make them unsuitable for clinical application, they are useful for investigating the underlying biology (Fig. 1A). Compounds previously investigated in this laboratory and others include the following: ARDP0006, which inhibits NS2B/3 proteinase activity (22), especially at one intramolecular cleavage site within NS3 (23); ST-148, a compound that hyperstabilizes core protein interactions and has been shown to display reduced selection for resistant viruses due to the dominance of drug-susceptible genomes (24, 25); spautin-1, an inhibitor of cellular autophagy (26) which has been shown to disrupt virion maturation (6).

In this work, we aimed to identify therapeutics with potential for repurposing toward treatment of dengue virus. We used ARDP0006, ST-148, and spautin-1 as our queries to computationally search a highly curated library of safe-in-human compounds with the goal of identifying those with three-dimensional chemical similarity to our known antiviral query compounds (27). For each query compound, we identified a safe-in-human hit that strongly inhibited dengue virus replication in tissue culture and exhibited antiviral mechanisms similar to those of the initial query compound. This approach offers advantages compared to traditional screens, namely, the speed and cost benefits of *in silico* work and the preexistence of strong hypotheses for mechanisms of action based on the known functions of the previously studied compounds.

## RESULTS

### Selection of methodology and compound library for *in silico* screening.

To identify pharmaceuticals whose safety profiles in humans are already known, we utilized a chemical library termed SWEETLEAD (structures of well-curated extracts, existing therapies, and legally regulated entities for accelerated discovery) (27). SWEETLEAD is a highly curated *in silico* collection of thousands of FDA-approved compounds, medicinal herbs, and controlled substances with known activities. We applied a ligand-based virtual screening strategy based on the assumption that chemically and structurally similar compounds have similar biological activities (28). In particular, we used ROCS (Rapid Overlay of Chemical Structures) software (29), an algorithm for ligand-based virtual screening that compares three-dimensional (3D) shape and chemical similarity between pairs of molecules, to search the SWEETLEAD database for compounds with structural similarity to three inhibitors of dengue virus infection with known biochemical targets: ARDP0006, ST-148, and spautin-1 (Fig. 1B).

### Selection and initial testing of ROCS-identified compounds for antiviral activity.

For each query molecule, the top 500 compound hits obtained from the ROCS algorithm, as ranked by the Tanimoto combo score (see Data Set S1 in the supplemental material), were manually inspected for similar chemical and structural features. They were further prioritized by factors such as a lack of known side effects, commercial availability, and the intellectual property considerations that would be needed for worldwide, inexpensive use. Fourteen candidates were tested for their effects on dengue virus growth and toxicity in BHK-21 and Huh7 cells (see Table S1 and Fig. S1 in the supplemental material). Three that exhibited the strongest antiviral activities in cell culture without evidence of cytotoxicity were selected for further study: pyrimethamine (a hit of ARDP0006), niclosamide (a hit of ST-148), and vandetanib (a hit of spautin-1).

**FIG 2:**
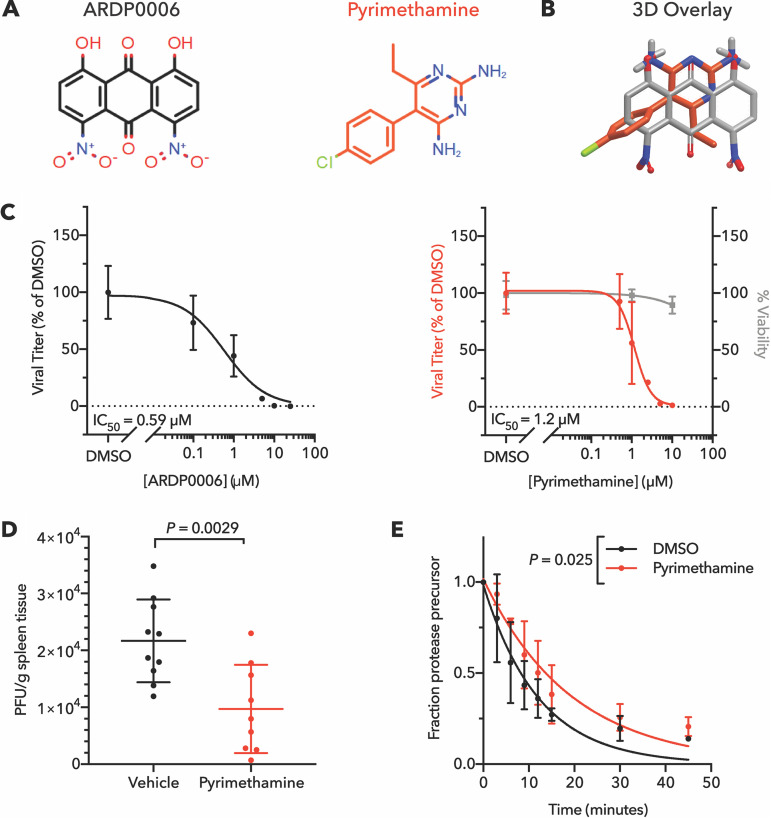
Pyrimethamine inhibits dengue virus and NS2B/3 proteinase cleavage like ARDP0006. (A) Chemical structures and (B) ROCS overlay of ARDP0006 and pyrimethamine. (C) Dose-response curves for ARDP0006 and pyrimethamine treatment of DENV2-infected Huh7 cells revealed IC_50_ values of 0.59 μM and 1.2 μM, respectively. Compounds were added 1 h prior to infection, removed, and then readministered after removal of viral inoculum. Infectious virus in cell supernatant was quantified by plaque assay at 24 hpi (left *y* axis, black or red). Cell viability was measured at the same pyrimethamine doses 24 h posttreatment by WST-1 assay (right *y* axis, gray). Viral titer and cell viability were normalized to DMSO-treated samples. (D) Splenic titers of DENV2-infected AGB6 mice at 4 days postinfection. Mice were infected with 1 × 10^6^ PFU DENV2 and treated twice daily with 20 mg/kg pyrimethamine (*n *= 9) or a vehicle control (*n *= 10). Data were analyzed by two-tailed unpaired *t* test. (E) Cleavage kinetics of NS2B/3/4A in the presence of 100 μM pyrimethamine. NS2B/3/4A protein was expressed *in vitro* and radiolabeled with [^35^S]methionine, followed by treatment with 100 μM pyrimethamine or DMSO. Reaction samples were obtained over a 45-min time course, separated by SDS-PAGE, and the fraction of uncleaved proteinase precursor remaining was determined as described previously (23). Data from three replicates were fit using single-phase exponential decay functions, and *P* value was determined by comparing the rate constant, *K*, of the models using the extra sum-of-squares F test.

**FIG 3:**
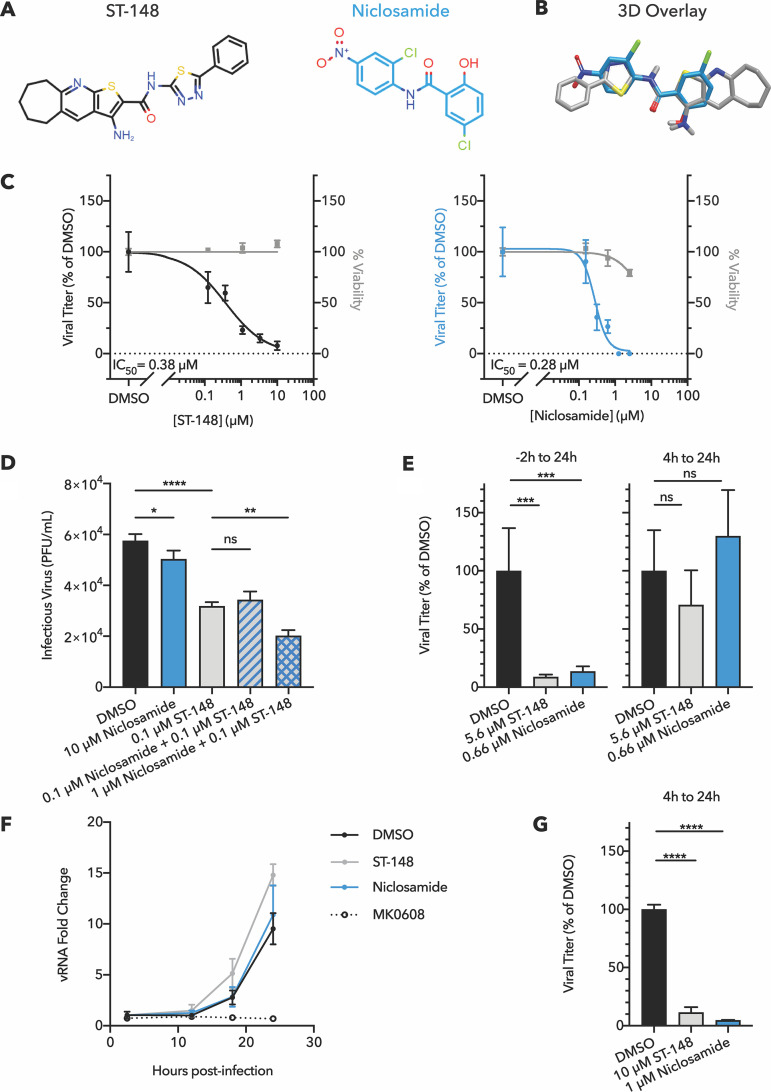
Niclosamide augments ST-148 virucidal activity and functions most potently early in the dengue infectious cycle. (A) Chemical structures and (B) ROCS overlay of ST-148 and niclosamide. (C) Dose-response curves for ST-148 and niclosamide treatment of DENV2-infected Huh7 cells revealed IC_50_ values of 0.38 μM and 0.28 μM, respectively. Compound addition, viral growth, and cytotoxicity assays were performed as described in the legend to Fig. 2C. (D) Virucidal activity of compounds at the concentrations indicated was measured after 60-min incubation of drugs with virus-containing solutions at 37°C. Viral titer was quantified by plaque assay on BHK-21 cells. *n *= 3. Statistical significance was evaluated by one-way ANOVA with Sidak’s multiple-comparison test and indicated as follows: *, *P* < 0.05; **, *P* < 0.01; ****, *P* < 0.0001; ns, not significant. (E) Infected Huh7 cells were treated with ST-148 and niclosamide at the IC_90_ doses for different durations, as indicated, within a single cycle of infection (24 h). On the left, cells were pretreated for 2 h prior to infection, compounds were removed during a 1-h infection at an MOI of 0.5 PFU/cell and then readministered after removal of viral inoculum. On the right, cells were similarly infected and compounds were first introduced 4 h after starting the infection. Cell supernatant was collected at 24 h postinfection (hpi), and infectious virus was quantified by plaque assay. *n *= 4, one-way ANOVAs with Dunnett’s multiple-comparison tests, ns, not significant (*P* > 0.05); ***, *P* < 0.001. (F) To test the effect of ST-148 and niclosamide on viral RNA replication, Huh7 cells were infected for 1 h at an MOI of 0.5 PFU/cell. Infections were allowed to proceed for 4 h to allow viral entry and the initiation of infection, after which compounds were administered at the IC_90_ doses of 5.6 μM ST-148 or 0.66 μM niclosamide. Intracellular RNA was collected at the time points indicated and quantified by qRT-PCR. Only the control for inhibited vRNA replication, 50 μM MK0608 treatment, significantly reduced vRNA load relative to the 1% DMSO vehicle control (*P* < 0.001 at 18 hpi, *P* < 0.0001 at 24 hpi). *N *= 4, two-way ANOVA with Dunnett’s multiple-comparison test. (G) When administered at an increased dose, ST-148 and niclosamide were effective during later, postentry stages. After 4 h of infection, 10 μM ST-148 or 1 μM niclosamide was added; extracellular virus was collected at 24 hpi and quantified by plaque assay. *N *= 3, one-way ANOVA with Dunnett’s multiple-comparison test, ****, *P* < 0.0001.

10.1128/mBio.02839-20.1 TABLE S1 Summary of hits for lead compounds ARDP0006 and ST-148 screened for anti-dengue activity in tissue culture cells. Hit compounds were tested for anti-dengue activity and cell toxicity in tissue culture cell lines. Antiviral activity is summarized as follows: −, not antiviral, with titer greater than 50% of vehicle; +, weakly antiviral, with titer between 20 and 50% of vehicle; ++, moderately antiviral, with titer between 5 and 20% of vehicle; +++, highly antiviral, with titer less than 5% of vehicle. Toxicity at these doses is represented as follows: −, nontoxic, with cell viability greater than 80% of vehicle; +, mild toxicity, with cell viability between 60 and 80% of vehicle; +++, high toxicity, with cell viability less than 60% of vehicle. Data summarized here are depicted graphically in Fig. S1 and Fig. 2C, 3C, and 4C. The Tanimoto combo score of each hit during initial ROCS screening is indicated; higher Tanimoto combo scores are indicative of increased chemical similarity. Download Table S1, PDF file, 0.04 MB. Copyright © 2020 Moshiri et al. 2020 Moshiri et al. This content is distributed under the terms of the Creative Commons Attribution 4.0 International license.

10.1128/mBio.02839-20.3 FIG S1 Initial screening of eliminated hit compounds for anti-dengue activity in tissue culture cells. BHK-21 cells were pretreated with the compounds at the indicated concentrations for 30 min, infected with dengue virus at an MOI of 0.1 PFU/cell for 1 h, and then posttreated with compound. At 48 hpi, cellular supernatant was collected and titered by plaque assay (purple). Cell viability in uninfected cells in the presence of compounds for 48 h was measured by WST assay (gray). Both viral titer and cell viability are normalized to their relative DMSO vehicle control, *N = *2. Download FIG S1, PDF file, 0.06 MB. Copyright © 2020 Moshiri et al. 2020 Moshiri et al. This content is distributed under the terms of the Creative Commons Attribution 4.0 International license.

10.1128/mBio.02839-20.6 DATA SET S1 ROCS *in silico* screening results for query compounds ARDP0006, ST-148, and Spautin-1. 3D conformers of the query molecules were compared with the chemical structures in the SWEETLEAD database using ROCS. This data set contains the top 500 hits for each query, as ranked by Tanimoto combo score, which summarizes both the shape and chemical similarity. Compounds evaluated for anti-dengue activity in tissue culture are highlighted in yellow, and those demonstrated to have similar antiviral mechanisms to the relative query compound are highlighted in green. Download Data Set S1, XLSX file, 0.6 MB. Copyright © 2020 Moshiri et al. 2020 Moshiri et al. This content is distributed under the terms of the Creative Commons Attribution 4.0 International license.

### Pyrimethamine inhibits NS2B/3 proteinase cleavage.

The NS2B/3 proteinase of dengue virus cleaves itself and other nonstructural viral proteins out of the nascent polyprotein and is necessary both for efficient polyprotein processing and cleaving host restriction factors (30, 31). In the presence of NS2B/3 proteinase inhibitor ARDP0006, we have presented evidence that inhibition of obligately intramolecular self-cleavage events results in accumulation of uncleaved or partially cleaved polyprotein precursors that are toxic to viral replication (23). Some of this evidence includes the fact that inhibition of viral growth in cell culture by this compound (50% inhibitory concentration [IC_50_] = 2.7 μM) (32) is much more potent than its inhibition of NS2B/3 proteinase cleavage activity in solution (IC_50_ = 620 μM), an observation we attribute to the accumulation of such toxic precursors (23). We found that pyrimethamine, an anti-malarial FDA-approved drug, shares 3D chemical structural similarity with ARDP0006 (Fig. 2A). This similarity includes the overlap of core aromatic rings of the two compounds as well as overlap between a pair of hydroxyl groups in ARDP0006 with a pair of primary amine groups on pyrimethamine (Fig. 2B). Therefore, we sought to determine whether pyrimethamine inhibits dengue virus growth by a mechanism similar to that of ARDP0006.

To characterize pyrimethamine’s antiviral activity, we evaluated viral titer in the presence of increasing drug concentrations. The IC_50_ of viral inhibition in a single infectious cycle was 1.2 μM (Fig. 2C), only twofold higher than the IC_50_ of ARDP0006 observed here (0.59 μM [Fig. 2C]). Unlike ARDP0006, pyrimethamine is well tolerated in healthy mice (Table S2), and thus, it could be tested for dengue virus inhibition *in vivo*. Dengue virus growth and pathogenesis were monitored in a standard mouse model, in which C57BL/6 mice that do not express either the type 1 or type 2 interferon receptors (AGB6 mice) are infected intravenously. A significant reduction in splenic titer of dengue virus 4 days after infection was observed, consistent with the antiviral effect observed in cultured cells (Fig. 2D). We attempted to perform survival experiments, but sustained treatment with pyrimethamine caused marked mortality in infected mice. We attribute this increased mortality to anemia, which is a well-described side effect of pyrimethamine and would be expected to worsen dengue pathology. Since direct-acting antivirals are most effective early in the course of infection, this compound would likely be used prior to the development of serious symptoms in a clinical setting and therefore could still represent a useful therapeutic. However, any clinical use of pyrimethamine during a hemorrhagic phase of dengue infection would clearly need to address such side effects.

10.1128/mBio.02839-20.2 TABLE S2 Acute toxicity of computationally identified, FDA-approved hit compounds. Toxicity for hits identified in the SWEETLEAD database as having chemical similarity to query molecules as reported in the PubChem database. Data for oral and intraperitoneal administration are reported, where available. Fifty percent lethal dose (LD_50_) values represent acute toxicity for a single dose by the relevant delivery route. Download Table S2, PDF file, 0.05 MB. Copyright © 2020 Moshiri et al. 2020 Moshiri et al. This content is distributed under the terms of the Creative Commons Attribution 4.0 International license.

To determine whether pyrimethamine inhibits intramolecular NS2B/3 proteinase activity like ARPD0006, we tested its effect on the cleavage of NS2B/3/4A precursor in protein translation extracts. This *in vitro* translation-based proteinase cleavage assay demonstrated that pyrimethamine significantly slowed dengue virus NS2B/3 cleavage but, like ARDP0006, required a dose far greater than the antiviral IC_50_ to achieve this effect (Fig. 2E). The striking difference between cell culture IC_50_ and biochemical IC_50_ suggests that pyrimethamine inhibits dengue virus in a manner similar to the query compound ARDP0006 by altering the proteolytic cleavage activity of NS2B/3 in a way that results in accumulation of uncleaved viral precursors that are toxic to essential viral processes in infected cells. We conclude that pyrimethamine does not require complete efficacy of inhibiting proteolytic activity *per se*, but rather even minor inhibition of proteinase activity can lead to effective dampening of infection.

### Niclosamide augments virucidal activity of query compound ST-148.

The core protein of dengue virus is an attractive target for antiviral drug development due to its complex oligomerization state, making it a candidate for dominant drug targeting (17). The effect of query compound ST-148 is to hyperstabilize protein-protein interactions between monomers of core protein in the viral capsid, leading to inhibition of the early stage of viral uncoating as well as later assembly steps (24, 25). We identified niclosamide, an anti-helminthic drug, as having structural similarity to ST-148 (Fig. 3A). Figure 3B shows that niclosamide and ST-148 display overlap of both the central aromatic rings and the amide bonds. Additionally, there is good overlap between the primary amine group in ST-148 and the hydroxyl group in niclosamide.

The ability of niclosamide to inhibit dengue virus infection was tested in single-cycle growth curves (Fig. 3C). The IC_50_ for niclosamide inhibition of infectious extracellular virus production (0.28 μM) was comparable to that observed for ST-148 (0.38 μM) and identical to a previously reported value for Zika virus (0.28 μM [33]). Capsid-binding viral inhibitors can be directly virucidal, inhibit viruses through cellular mechanisms such as entry and assembly, or both. To test whether ST-148 or niclosamide is directly virucidal, infectious virus stocks were incubated in the presence of drugs or dimethyl sulfoxide (DMSO) controls for 1 h at 37°C, after which viral infectivity was quantified by plaque assay in the absence of compounds. We observed that coincubation of virions with just 0.1 μM ST-148 significantly reduced viral infectivity (Fig. 3D). At a much higher concentration, niclosamide was also significantly, albeit slightly, virucidal. However, addition of 1 μM niclosamide to 0.1 μM ST-148 significantly increased the virucidal effect of ST-148. Thus, we hypothesize that niclosamide, like ST-148, binds directly to the viral core protein. The differences in the steepness of the inhibition curves (Fig. 3C) further suggest that niclosamide binds more cooperatively to virions than ST-148.

Previous reports on the inhibition of dengue virus by niclosamide have argued for several vulnerable points in the viral infectious cycle: at an early step, preventing the endosomal acidification required for uncoating; at a protein-processing step, by inhibiting NS2B/3 proteinase activity; and at a later step, by interfering with virion maturation (34–36). To test a requirement for ST-148 and niclosamide at early times in infection, which would include direct action on virion infectivity as well as the previously postulated endosomal maturation, we compared the effects of IC_90_ doses when the compounds were present for the full time course of infection or administered at 4 h after infection to allow early steps of viral entry and uncoating to occur. Delayed addition of either compound eliminated the observed reduction of viral titer (Fig. 3E), indicating that, at this low concentration, strong inhibition occurred only during the first 4 h of infection. We also explicitly tested the effect of both ST-148 and niclosamide on the accumulation of viral RNA, which would be reduced if translation, protein processing, or RNA synthesis were inhibited. When either compound was added at 4 h postinfection at its IC_90_ dose, no difference in viral RNA abundance was observed, whereas the addition of MK0608, a potent inhibitor of viral RNA synthesis, strongly reduced viral RNA abundance (Fig. 3F). Thus, we conclude that the most critical effects of both ST-148 and niclosamide occur early in the viral infectious cycle, during the process of receptor binding, cell entry, or genome release. Niclosamide’s antiviral activity during early infection has been previously attributed to blocking endosomal acidification; however, drug binding to core protein should affect this stage as well, as a virion with hyperstabilized core protein interactions would be incapable of uncoating. Given that previous literature has suggested that niclosamide can target late stages of viral replication, we increased the doses of niclosamide and ST-148 administered after the entry and uncoating steps. At the increased doses, both compounds inhibited virion production (Fig. 3G), confirming that further inhibitory effects of niclosamide on later stages in the infectious cycle such as protein processing, virion assembly, and acidification-dependent virion maturation could also contribute to its antiviral efficacy (36).

### Vandetanib and query compound spautin-1 disrupt viral egress independent of the autophagy-inducing VPS34 complex.

We and others have observed that dengue virus growth is greatly enhanced by components of the cellular autophagy pathway (6, 37, 38). Autophagy components that enhance dengue virus infection include VPS34, a kinase present in complex with Beclin-1 and needed for initiation of canonical autophagy; ATG9, which is involved in lipid acquisition; and LC3, which is needed for cargo loading and induction of membrane curvature. Other canonical autophagy factors, specifically ULK1, Beclin-1, and ATG5 are dispensable during dengue virus infection (38).

**FIG 4:**
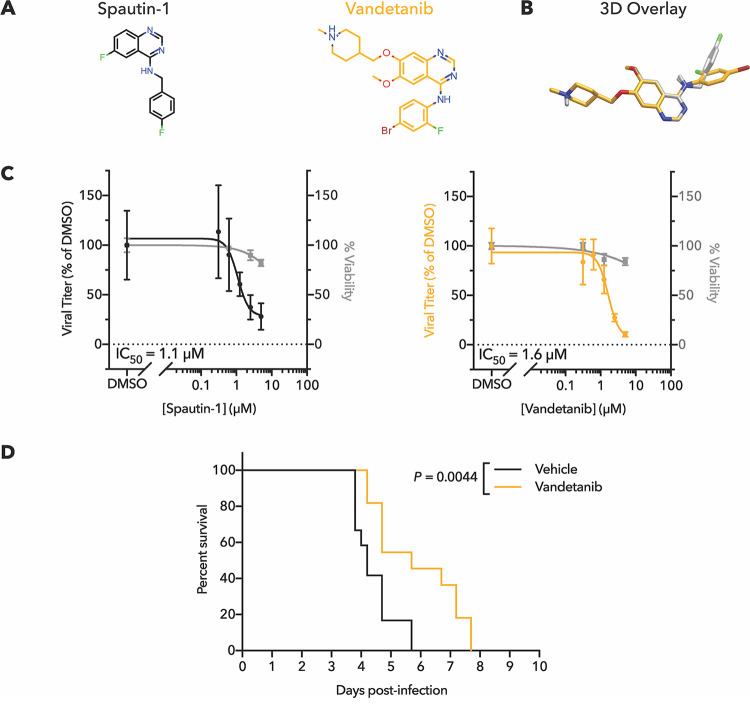
Vandetanib inhibits dengue virus in Huh7 cells and extends survival in infected mice. (A) Chemical structures and (B) ROCS overlay of spautin-1 and vandetanib. (C) Dose-response curves for spautin-1 and vandetanib for treatment of DENV2-infected Huh7 cells revealed IC_50_ values of 1.1 μM and 1.6 μM, respectively. Compound addition, viral growth, and cytotoxicity assays were performed as described in the legend to Fig. 2C. (D) Administration of 15 mg/kg vandetanib twice daily significantly extended survival of DENV2-infected AGB6 mice. Log rank (Mantel-Cox) test, *P* = 0.0044. *N *= 12 (vehicle treated) or *N *= 11 (vandetanib treated).

The most potent small-molecule inhibitor of the early stages of autophagy is spautin-1 (specific and potent autophagy inhibitor 1), which we have previously found to inhibit dengue virus growth by disrupting viral maturation and release (6). When spautin-1 was used as a query compound, ROCS analysis of the SWEETLEAD database identified an FDA-approved drug, vandetanib (Fig. 4A), as having structural similarity. Vandetanib, a multireceptor tyrosine kinase inhibitor, is approved for the treatment of medullary thyroid cancer. Figure 4B shows that spautin-1 and vandetanib share a central scaffold and that there is some overlap of the side benzene rings. In a dose-response assay, vandetanib inhibited dengue infection in Huh7 cells with an IC_50_ of 1.6 μM (Fig. 4C), comparable to that of spautin-1 (1.1 μM). Furthermore, vandetanib was only mildly toxic (Table S2), allowing *in vivo* testing in mice susceptible to dengue virus pathogenesis. Oral dosing of dengue virus-infected mice showed that vandetanib significantly extended survival following dengue infection (Fig. 4D). Using the same mouse model and dosing regimen, however, we observed that vandetanib treatment did not significantly impact splenic viral titer in infected mice (Fig. S2). In humans, slow-release versions have been formulated to optimize dose and delivery; it is possible that such optimization would be needed in mice as well to reveal any effects on viral growth.

10.1128/mBio.02839-20.4 FIG S2 Vandetanib treatment in dengue-infected mice did not affect splenic titers. AGB6 mice were infected with 3 × 10^5^ (light pink), 4.1 × 10^5^ (magenta), or 1 × 10^6^ (purple) PFU dengue virus. Mice were treated twice daily with 15 mg/kg (30 mg/kg per day) vandetanib or drug vehicle alone, beginning 4 h prior to infection. Splenic titer at 4 days postinfection is shown. Data were analyzed by two-tailed, unpaired *t* test. Download FIG S2, PDF file, 0.07 MB. Copyright © 2020 Moshiri et al. 2020 Moshiri et al. This content is distributed under the terms of the Creative Commons Attribution 4.0 International license.

**FIG 5:**
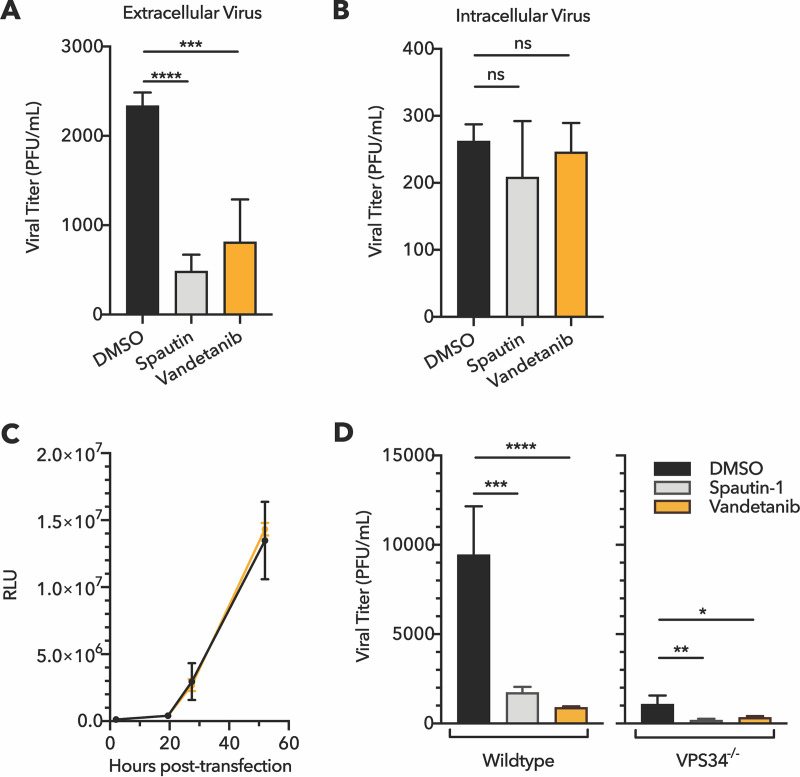
Vandetanib and spautin-1 inhibit dengue maturation and egress in a VPS34-independent manner. Comparison of (A) extracellular and (B) intracellular viral titer at 24 hpi during treatment at the IC_90_ doses of spautin-1 (2.4 μM) or vandetanib (3.6 μM) revealed abundant intracellular virus despite reduced extracellular virus in the presence of each compound. Cells were pretreated for 1 h, infected for 1 h at an MOI of 0.5 PFU/cell, and posttreated for an additional 23 h. *N *= 4, one-way ANOVA with Dunnett’s multiple-comparison test. ns, not significant (*P* > 0.05); **, *P* < 0.01; ****, *P* < 0.0001. (C) Huh7 cells transfected with a luciferase-expressing RNA replicon were treated with 3 μM vandetanib (orange) or 1% DMSO vehicle control (black) for 1 h prior to transfection and up to 52 h posttransfection. Luciferase intensity was quantified as a measure of viral protein abundance. Luciferase intensity was shown as relative light units (RLU). The use of an RNA replicon that is directly transfected into cells reflects only translation and RNA replication steps of viral infection, not viral packaging or cell exit. *N *= 4, two-way ANOVA with Sikak’s multiple-comparison test. Statistical significance was defined as *P* < 0.05; no significant differences were observed between DMSO and vandetanib treatments at any time point. (D) VPS34 was not required for spautin-1 or vandetanib anti-dengue activity. Wild-type and VPS34 knockout Huh7 cells were treated with 5 μM spautin-1, 5 μM vandetanib, or DMSO. Cells were pretreated, infected at an MOI of 0.5, and posttreated as described above for panels A and B. At 24 hpi, cellular supernatant was collected and titered by plaque assay. *N ≥ *3. One-way ANOVA with Dunnett’s multiple-comparison test; *, *P* < 0.05; **, *P* < 0.01; ***, *P* < 0.001; ****, *P* < 0.0001.

To test whether vandetanib, like spautin-1, inhibited only post-RNA replication stages of dengue growth, we compared the amount of extracellular virus (Fig. 5A) with intracellular virus (Fig. 5B). After 24 h of infection in the presence of spautin-1 or vandetanib at their calculated IC_90_ doses (2.4 μM and 3.6 μM, respectively), virus in the extracellular supernatant was significantly reduced, as expected (Fig. 5A). However, after infected cells were washed, collected, and lysed by repeated freeze/thaw, we observed that the abundance of infectious intracellular virus was unchanged by spautin-1 or vandetanib treatment (Fig. 5B). These results reveal that both spautin-1 and vandetanib inhibit dengue virus at the stage of viral egress at this dosage. To determine whether earlier steps of viral protein synthesis and RNA amplification were truly unaffected by vandetanib, we tested the effect of the drug on the amplification of dengue virus replicon RNA. When a dengue virus 2 (DENV-2) luciferase-expressing replicon was delivered by RNA transfection into Huh7 cells, dosing with vandetanib had no effect on luciferase production. This indicates that replicon RNA synthesis and protein expression were not impacted (Fig. 5C), consistent with an effect of the drug at only the latest stages of viral growth.

Given the findings that spautin-1 represses autophagy by causing degradation of the VPS34/Beclin-1 complex (26) and that VPS34 is required for efficient virus production while other components of this complex are not (38), we hypothesized that spautin-1 and vandetanib both required VPS34 to inhibit dengue virus via the autophagy pathway. To investigate this, we compared antiviral activity of spautin-1 and vandetanib in wild-type Huh7 cells and in CRISPR/Cas9-generated VPS34 knockout Huh7 cells (Fig. 5D; Fig. S3). As expected, VPS34 knockout significantly reduced viral titer, even in the absence of spautin-1 or vandetanib. However, both compounds retained significant antiviral activity in VPS34 knockout cells, indicating that the molecular mechanism of these compounds is not exclusively mediated by VPS34. These results argue that vandetanib, like spautin-1, inhibits dengue virus egress by an unknown mechanism that may differ from the effect of spautin-1 on autophagy.

10.1128/mBio.02839-20.5 FIG S3 Validation of CRISPR/Cas9 VPS34 knockout (KO) Huh7 cells by immunoblotting and Sanger sequencing. VPS34 was knocked out in a Huh7 background through insertion of 4 bp in exon 3, as described previously (E. Abernathy, R. Mateo, K. Majzoub, N. van Buuren, et al., PLoS Biol 17:e2006926, 2019, https://doi.org/10.1371/journal.pbio.2006926). (A) Protein lysates from wild-type Huh7 and two separate VPS34 KO clones (clones 5 and 6) were obtained, separated by SDS-PAGE, and immunoblotted using antibodies targeted to VPS34 and GAPDH. Efficient knockout of VPS34 protein expression was observed in clone 6. (B) Genomic DNA from VPS34 clone 6 KO cells was isolated, the VPS34 region was PCR amplified around the targeted cut site, and the PCR product was sequenced by Sanger sequencing to ensure incorporation of the 4-bp insertion (highlighted in blue) by the guide RNAs. VPS34 knockout of clone 6 was determined to be successful, and this clone was utilized in further experiments. Download FIG S3, PDF file, 0.1 MB. Copyright © 2020 Moshiri et al. 2020 Moshiri et al. This content is distributed under the terms of the Creative Commons Attribution 4.0 International license.

## DISCUSSION

With no antiviral treatments currently available for patients suffering from dengue virus infection, there is a great unmet need for therapeutics targeting this disease. Given that the cost of drug discovery continues to increase (19), efficient approaches to develop such therapeutics are urgently needed. In this study, we used a ligand-based virtual screening tool to compare the three-dimensional chemical similarity of anti-dengue research compounds with safe-in-human drugs in the SWEETLEAD database and identified three FDA-approved drugs that inhibit dengue virus from this database: pyrimethamine, niclosamide, and vandetanib.

Pyrimethamine, a folic acid antagonist used against apicomplexan parasitic infections, shares structural similarity to query compound ARDP0006, an inhibitor of the dengue NS2B/3 proteinase. Pyrimethamine reduced the dengue virus titer with an IC_50_ of 1.2 μM and significantly reduced viral splenic burden in infected mice (Fig. 2D). Pyrimethamine was previously found in a high-throughput infectivity screen to inhibit Zika virus growth, but no potential mechanism has been identified (39). We found that, like query compound ARDP0006, pyrimethamine affected the kinetics of intramolecular NS2B/3 cleavage, slowing proteinase cleavage in translation extracts. These results argue that pyrimethamine inhibits dengue virus, and possibly Zika virus, through inhibition of the NS2B/3 viral proteinase. Our previous work with ARDP0006 argued that the strong inhibition of viral growth by the relatively modest NS2B/3 proteinase inhibition in solution results from the accumulation of improperly cleaved viral products (23).

Niclosamide, an FDA-approved drug used to treat tapeworm infections, was identified here via its similarity to ST-148 and shown to significantly inhibit dengue virus growth, with an IC_50_ of 0.28 μM (Fig. 3C). Niclosamide has been previously published as an inhibitor of both dengue and Zika viruses (33–36) and has been demonstrated to exert many additional therapeutic effects, including inhibition of other viral and bacterial infections, cancer, and metabolic disease (33, 39–46). Our hypothesis was that niclosamide, like the query compound ST-148, might function specifically as an inhibitor of core protein function. Indeed, we show here that niclosamide can augment the virucidal activity of ST-148 and that the inhibitory effects of low concentrations of niclosamide and ST-148 are realized within the first few hours of infection. This sensitive early step is likely to be the uncoating of the viral capsid, which direct binding of these compounds to core protein would prevent, but may also reflect a defect in endosomal acidification (35). At higher concentrations, niclosamide is inhibitory at later stages of infection as well. This could also result from a direct effect on core protein in viral assembly or on steps such as NS2B/3 proteinase function (34) and the acidification of membrane compartments required for virion maturation (36). *In vivo*, niclosamide has been previously shown to inhibit dengue virus growth and pathogenesis in a suckling mouse model of infection (35). Should niclosamide truly inhibit viral growth at multiple stages, this could contribute to its effectiveness and would likely provide a high genetic barrier to antiviral resistance.

Last, we demonstrated inhibition of dengue virus growth by vandetanib, a multiple-receptor tyrosine kinase inhibitor approved for use against medullary thyroid cancer. For both vandetanib and its query compound spautin-1, no inhibition of any step up to and including the production of intracellular infectious virus was observed; however, the amount of extracellular infectious virus produced was inhibited at the IC_90_ concentrations. Thus, at low drug concentrations, both vandetanib and query compound spautin-1 inhibit dengue virus at the stage of viral egress. Given that spautin-1 is an inhibitor of cellular autophagy and that virion assembly and maturation depend on virally induced membranous structures that require autophagy components, we investigated the effect of autophagy protein VPS34 on the inhibition of virion production by spautin-1 and vandetanib. Spautin-1 reportedly inhibits autophagosomal initiation and maturation by inhibiting deubiquitylases USP10 and USP13, which stabilize the Beclin-1/VPS34 complex (26). However, even though VPS34 is required for efficient growth of dengue virus (38), we discovered that both vandetanib and spautin-1 retained their ability to limit dengue virus egress in the absence of VPS34. Thus, there are other players in the inhibition of dengue virus that may be shared by spautin-1 and vandetanib.

Vandetanib’s inhibition of viral egress could theoretically be related to its known ability to inhibit receptor tyrosine kinases such as epidermal growth factor receptor (EGFR) and vascular endothelial growth factor receptor (VEGFR), which is why it is FDA approved to treat certain cancers. Spautin-1 has also been shown to inhibit EGFR signaling (47). EGFR and other receptor tyrosine kinases, which are known to modulate intracellular vesicle trafficking, are targeted by kinase inhibitors erlotinib and sunitinib, which are also known inhibitors of dengue virus (48). However, these inhibitors function during the stage of viral entry rather than egress, making it unlikely that EGFR signaling is the most important target of the direct antiviral activity of spautin-1 and vandetanib.

It is possible, however, that vandetanib inhibition of EGFR or VEGFR *in vivo* may alleviate some of the pathology of dengue hemorrhagic fever and dengue shock syndrome; VEGFR signaling directly induces vasodilation (49). During severe dengue, patients experience increased vascular permeability, plasma leakage, and reduced platelet count (50–52). VEGF levels are significantly elevated in plasma from patients with severe dengue fever compared to patients with uncomplicated dengue or healthy controls (53, 54). Similarly, recent studies have argued that EGFR inhibition may also lead to suppressed pathology of dengue infection (55). Clinical studies have correlated severe dengue disease with reduced abundance of prostasin, which reduces EGFR expression; further, EGFR knockout reduced dengue pathogenesis in a suckling mouse model (55). Therefore, vandetanib inhibition of VEGFR-induced vasodilation or EGFR-mediated alterations in cell physiology may alleviate hemorrhagic symptoms caused by severe dengue. The possibility of directly inhibiting viral egress and simultaneously alleviating pathology of severe dengue makes vandetanib an exciting candidate for translation into a clinical setting.

When it comes to the development of antiviral drug resistance, not all targets are equal. By strategically selecting candidates with higher genetic barriers to resistance, we can develop antivirals that are more effective and have longer durations of clinical utility. As a host-targeting compound, antiviral resistance to vandetanib is unlikely to be rapidly selected among an existing virus population (16, 56). The target of query compound ST-148 and, most likely, its hit compound niclosamide is core, a viral protein for which drug susceptibility has been shown to be genetically dominant (57). Specifically, a single monomer of dengue core protein must assemble with other core molecules to form a larger oligomer, the capsid shell. When, in the presence of drug, a single viral genome within the cell randomly arises which is drug resistant, the monomer produced will be incorporated into a larger oligomer that also contains drug-susceptible molecules, rendering the entire chimera drug susceptible. The target of query compound ARDP0006 and hit compound pyrimethamine is NS2B/3, which is likely to be a dominant drug target as well. This proteinase must first perform multiple intramolecular proximal cleavages in *cis* to free itself from the larger polyprotein before it can cleave at more distal viral junctions. Because these cleavages occur only in *cis*, a drug-resistant proteinase would not be able to rescue a drug-susceptible neighbor in *trans*, leading to accumulation of uncleaved precursors that interfere with outgrowth of all viruses in the cell, even those with drug-resistant genomes (23). Thus, for all three anti-dengue compounds described here, the outlook is promising because each functions through a target for which the development of drug resistance is disfavored.

Tool compounds that are unsuitable for use in humans have been identified for many infectious agents. Structure-based *in silico* screening is emerging as a powerful technique to quickly identify candidates for drug development for many indications, including viral diseases (58–60). The workflow used here can be easily adapted for a wide range of biological indications for which a query compound has been identified. The SWEETLEAD database of safe-in-human compounds is freely available to download in a variety of formats (27) and contains high-confidence, curated structural information for 4,442 approved drugs, controlled substances, and herbal isolates. Once downloaded, the SWEETLEAD database can be searched using any computational screening workflow desired, including the ROCS virtual screening software utilized in this study (29). Utilizing these computational tools to conduct *in silico* primary screening drastically reduced the number of compounds we were required to test in tissue culture, expediting the process of identifying candidates for repurposing and providing strong, testable hypotheses about their mechanisms. As FDA-approved drugs with antiviral mechanisms that likely disfavor drug resistance, pyrimethamine, niclosamide, and vandetanib are all promising candidates for repurposing against dengue virus that should be further investigated to meet this pressing global public health need.

## MATERIALS AND METHODS

### Computational screening.

Three query molecules were used for *in silico* ligand-based screening: ST-148, ARDP0006, and Spautin-1. 3D conformers of the molecules were generated using OMEGA (version 2.5.1.4, OpenEye Scientific Software, Santa Fe, NM) (61) and then compared with the chemical structures in the SWEETLEAD database (https://simtk.org/projects/sweetlead) (27), using ROCS (version 3.2.1.4, OpenEye Scientific Software, Santa Fe, NM) (29). The top 500 hits for each query were ranked by Tanimoto combo score, which summarizes both the shape and chemical similarity, and manually inspected. Six compounds were selected for ARDP0006, seven compounds were selected for ST-148, and two compounds were selected for spautin-1. Compounds were purchased from chemical vendors (AK Scientific, Santa Cruz Biotech, and Sigma-Aldrich) for experimental validation.

### Viruses and cell culture.

Dengue virus strains 16681 (used in cell culture, gift of Richard Kinney) and PL046-2M (used in mouse experiments, gift of Eva Harris) (62) were produced as previously described (23). BHK-21 cells were grown at 37°C with 5% CO_2_ in Dulbecco modified Eagle medium (DMEM) (HyClone, GE Healthcare Life Sciences) supplemented with 10% bovine serum and 1 U/ml penicillin/streptomycin. Huh7 cells were grown at 37°C with 5% CO_2_ in DMEM supplemented with 10% fetal bovine serum, 0.1 mM nonessential amino acids (Gibco), 1 mM sodium pyruvate (Gibco), and 1 U/ml penicillin/streptomycin (Gibco). Huh7 CRISPR/Cas9 VPS34 knockout cells were generated as previously described (38) and validated by Sanger sequencing and immunoblotting (see Fig. S3 in the supplemental material).

### Plaque assays.

Dengue plaque assays were carried out on BHK-21 cells in 24-well plates. Cells were infected with 10-fold serial dilutions of virus, which were allowed to adsorb for at least 1 h at 37°C, and overlaid with standard cell culture medium plus 0.37% (w/vol) sodium bicarbonate and 0.8% (w/vol) Aquacide II (Millipore Calbiochem). After 7 days of incubation at 37°C, 5% CO_2_, cells were fixed with 5% (final) formaldehyde and stained with crystal violet for plaque enumeration.

### Assays for antiviral activity.

Between 4 × 10^4^ and 1 × 10^5^ Huh7 cells per well were seeded into 24-well plates 1 day prior to infection. Compounds were diluted to 100× final concentration in DMSO and then further diluted 1:100 into appropriate cell culture. Unless otherwise indicated, tested compounds were present for 1 h pretreatment and readministered for posttreatment following infection. Specifically, cells were pretreated with compounds or 1% DMSO for 1 h prior to infection. Media and drugs were removed from cells for infection at a multiplicity of infection (MOI) of 0.1 or 0.5 in 200 μl cold medium per well. After 1 h at 37°C, virus inoculum was removed by aspiration and replaced with medium plus compound or control. After 24-h total infection, infectious virus was quantified by plaque assay. Antiviral IC_50_ dose-response curves were fitted in GraphPad Prism 8 using the “[inhibitor] versus response, variable slope” function with a constraint “bottom > 0.” IC_90_ values are defined as the dose at which “*y =* (span × 0.1) + bottom.”

### Cell viability assays.

Mock-infected wells were used to determine cell viability in the presence of chemical compounds compared to 1% DMSO vehicle control. Cell viability was determined using the WST-1 kit (Abcam) per the manufacturer’s instructions. The 50% cytotoxic concentration (CC_50_) was calculated by fitting dose-response curves in GraphPad Prism 8 using the “[inhibitor] versus normalized response, variable slope” function.

### Virucidal activity assays.

Compounds were diluted to 100× in DMSO, then further diluted 1:100 into phosphate-buffered saline (PBS) containing dengue virus to a final volume of 200 μl and mixed. Aliquots were taken for plaque assays immediately after addition of compounds (time [*T*] = 0 min) or after incubation at 37°C for 60 min (*T* = 60 min).

### Intracellular viral RNA isolation and quantification.

Supernatants were removed from infected cells, and cells were washed with high-salt buffer (1 M NaCl, 50 mM sodium bicarbonate [pH 9.5]) for 3 min 4°C to remove cell-associated virus (63). RNA was extracted with TRIzol reagent (Thermo Fisher), precipitated with isopropanol, washed with 75% (vol/vol) ethanol, and resuspended in water. Positive-sense viral RNA (vRNA) and glyceraldehyde-3-phosphate dehydrogenase (GAPDH) transcripts were measured by quantitative reverse transcription-PCR (qRT-PCR) using the QuantiTect SYBR green RT-PCR kit (Qiagen) on an Applied Biosystems 7300 machine. Fold change in dengue viral RNA was calculated using the 2^−ΔΔ^*^Ct^* method and are relative to the early time point 1% DMSO control. Primer sequences used for GAPDH and viral genomic RNA are as follows (in the 5′ to 3′ orientation): GAPDH forward, CTGAGAACGGGAAGCTTGT; GAPDH reverse, GGGTGCTAAGCAGTTGGT; dengue virus (DENV) NS3A forward, AATGGGTCTCGGGAAAGGAT; DENV NS3A reverse, AAGAGCTGCTGTGAGAGTTA.

### Proteinase cleavage assays.

Proteinase cleavage assays were performed as previously described (23). Briefly, dengue protein NS2B/3/4A constructs were expressed in rabbit reticulocyte lysate (T_N_T coupled T7; Promega Bio Systems) programmed with 1 μg DNA/50 μl and labeled with 20 μCi of l-[^35^S]methionine (EasyTag; Perkin Elmer). Reaction mixtures were incubated at 30°C for 30 min before adding l-methionine (1 mM final) and DMSO (2% final) or pyrimethamine in DMSO. At the indicated times, 3-μl aliquots were immediately diluted with Laemmli sample buffer (4 volumes, 1× final), denatured (60°C, 10 min), and separated by sodium dodecyl sulfate-polyacrylamide gel electrophoresis (SDS-PAGE). Gels were dried under vacuum (80°C, 120 min) and exposed to a low-energy phosphor storage screen (Molecular Probes). Protein quantification was performed using ImageQuant TL 8.1 software (GE Healthcare Life Sciences).

### Replicon luciferase assays.

A dengue replicon derived from the dengue virus strain 16681 expressing *Renilla* luciferase and dengue nonstructural proteins has been previously established (7, 64). Huh7 cells were pretreated with compounds of interest or 1% DMSO in cell culture medium for 1 h. After pretreatment, 1 μg replicon RNA per well was transfected using Lipofectamine 3000 (Thermo Fisher Scientific) for 2 h at 37°C in opti-MEM (Gibco). Cells were washed three times after transfection with medium, then posttreated with medium plus compounds or 1% DMSO at 37°C. Transfected cells were harvested and analyzed using the *Renilla* luciferase assay system (Promega BioSystems). Luciferase intensity was measured with a GloMax luminometer (10-s signal integration; Promega BioSystems).

### Intracellular virus quantification.

Huh7 cells seeded into 24-well plates were pretreated with spautin-1 or vandetanib at their IC_90_ doses (2.4 μM and 3.6 μM, respectively) for 1 h at 37°C and infected with dengue virus at an MOI of 0.5. After 1 h, virus inoculum was aspirated and replaced with medium containing compounds or 1% DMSO. After 24-h total infection, extracellular virus was collected in cellular supernatant and quantified by plaque assay. To collect intracellular virus, cells were washed with PBS, incubated for 3 min at 4°C with high-salt buffer (1 M NaCl, 50 mM sodium bicarbonate [pH 9.5]) to detach cell-associated extracellular virus (63), washed twice with PBS, detached with 0.25% trypsin-EDTA, and isolated by centrifugation (500 × *g*, 5 min, 4°C). Infected cell pellets were resuspended in 750 μl cell culture medium, lysed by repeated freeze/thaw in liquid nitrogen followed by 37°C water bath. Debris was removed by centrifugation (3,200 × *g*, 5 min, 4°C), and infectious intracellular virus was quantified by BHK-21 plaque assay.

### Mice.

Based on the original mouse models of dengue virus infection in mice lacking type I and type II interferon signaling (65–67), C57BL/6 congenic mice lacking type I and type II interferon receptors (AGB6; C57BL/6.129-*Ifnar1*^tm1Agt^ *Ifngr1*^tm1Agt^) (57) were inoculated intravenously (IV) with dengue virus in 100 μl PBS plus 20% FBS at 8 to 12 weeks of age by retro-orbital injection under animal biosafety level 2 (ABSL2) conditions. Antiviral compound treatments were delivered by oral gavage twice daily and were begun 4 to 12 h prior to infection. Pyrimethamine was prepared in saline (0.9% NaCl) plus 2% (vol/vol) DMSO and 2% 2-hydroxypropyl-beta-cyclodextrin. Mice received 20 mg pyrimethamine per kilogram of body weight delivered twice daily (40 mg/kg per day). Vandetanib was prepared in saline plus 2% (vol/vol) DMSO, 2% (wt/vol) (2-hydroxypropyl)-beta-cyclodextrin in PBS. Infected mice received 15 mg vandetanib per kg weight, twice daily (30 mg/kg per day). Mice were euthanized at 4 days postinfection for determination of dengue virus quantities in splenic tissue by plaque assay. Mice were housed in an AAALAC-accredited mouse facility. Husbandry is performed in accordance with the *Guide for the Care and Use of Laboratory Animals*, 8th edition (68). Room conditions included a temperature of 23°C, relative humidity of 30% to 40%, and a 12-h:12-h light:dark cycle (lights on, 0700 h). All experiments involving mice were approved by the Institutional Animal Care and Use Committee of Stanford University under protocol APLAC-9296.
