# Supplementary material for: A Targeted Computational Screen of the SWEETLEAD Database Reveals FDA-Approved Compounds with Anti-Dengue Viral Activity
Source: mBio. 2020 Nov 10;11(6):e02839-20. doi: 10.1128/mBio.02839-20 (PMC7667029; doi:10.1128/mBio.02839-20)
Supplement: TABLE S1 [file mBio.02839-20-st001.pdf]

Lead Compound	Hit Compound	Antiviral	Toxicity	Tanimoto Combo
ARDP0006	Methoxsalen	−	−	0.959
	Triamterine	+++	+++	0.826
	Hydroxy tacrine	−	−	1.001
	Sulfisoxazole	+	+	0.830
	Pemirolast	−	−	0.875
	Pyrimethamine	+++	−	0.808
ST-148	Ondansetron	++	−	0.843
	Nifenazone	+	+	0.950
	Eltrombopag	−	−	0.926
	Meloxicam	−	+	0.972
	Doxazosin	+	+++	0.903
	Omeprazole	−	+	0.892
	Niclosamide	+++	−	1.002
Spautin-1	Vandetanib	++	−	0.914

Table S1. Summary of hits for lead compounds ARDP0006 and ST-148 screened for anti-dengue activity in tissue culture cells. Hit compounds were tested for anti-dengue activity and cell toxicity in tissue culture cell lines. Antiviral activity is summarized as: (−) not antiviral, with titer greater than 50% of vehicle; (+) weakly antiviral, with titer between 20-50% of vehicle; (++) moderately antiviral, with titer between 5-20% of vehicle; and (+++) highly antiviral, with titer less than 5% of vehicle. Toxicity at these doses are represented as: (−) nontoxic, with cell viability greater than 80% of vehicle; (+) mild toxicity, with cell viability between 60-80% of vehicle; and (+++) high toxicity, with as cell viability less than 60% of vehicle. Data summarized here are depicted graphically in Figs S1, 2C, 3C, and 4C. The Tanimoto combo score of each hit during initial ROCS screening is indicated; higher Tanimoto combo scores are indicative of increased chemical similarity.
