# Supplementary material for: A Targeted Computational Screen of the SWEETLEAD Database Reveals FDA-Approved Compounds with Anti-Dengue Viral Activity
Source: mBio. 2020 Nov 10;11(6):e02839-20. doi: 10.1128/mBio.02839-20 (PMC7667029; doi:10.1128/mBio.02839-20)
Supplement: FIG S1 [file mBio.02839-20-sf001.pdf]

Lead compound: ARDP0008
Target: NS2B/3 Protease

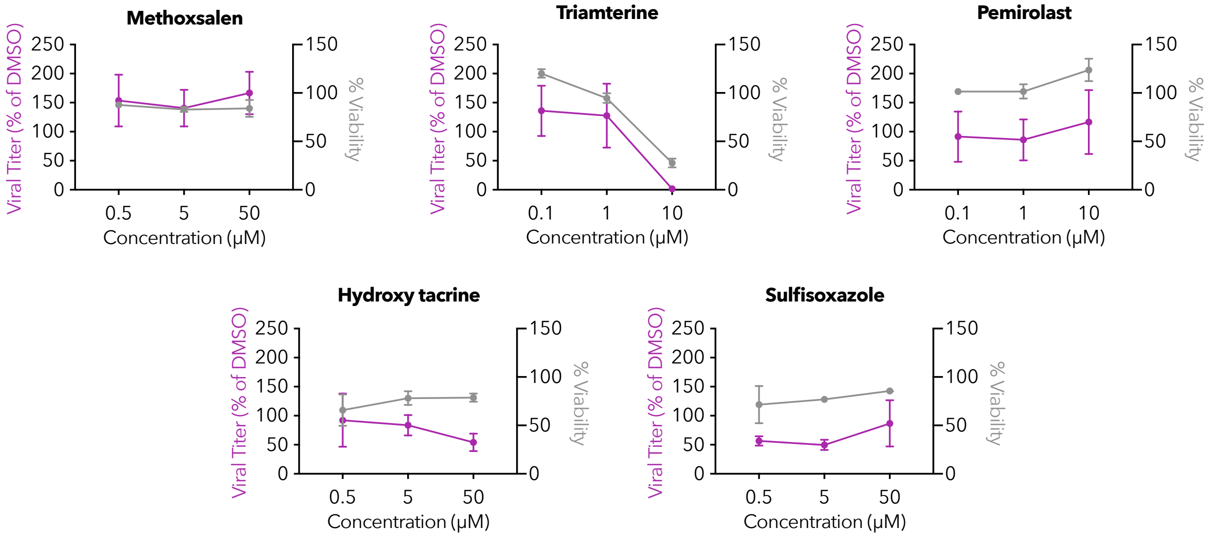

Lead compound: ST-148
Target: Core

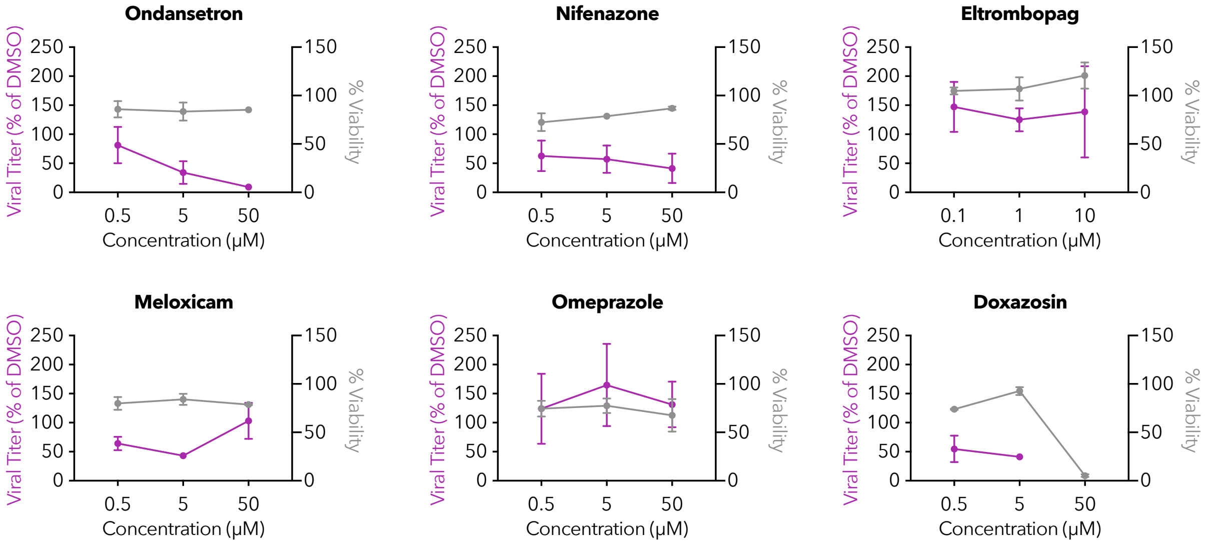

Fig S1. Initial screening of eliminated hit compounds for anti-dengue activity in tissue culture cells. BHK-21 cellls were pre-treated with the compounds at the indicated concentrations for 30 min, infected with dengue virus at MOI = 0.1 PFU/cell for 1 h, then post-treated with compound. At 48 hpi, cellular supernatant was collected and titered by plaque assay (purple). Cell viability in uninfected cells in the presence of compounds for 48 h was measured by WST assay (grey). Both viral titer and cell viability are normalized to their relative DMSO vehicle control, *N* = 2.
