# Supplementary material for: A Targeted Computational Screen of the SWEETLEAD Database Reveals FDA-Approved Compounds with Anti-Dengue Viral Activity
Source: mBio. 2020 Nov 10;11(6):e02839-20. doi: 10.1128/mBio.02839-20 (PMC7667029; doi:10.1128/mBio.02839-20)
Supplement: TABLE S2 [file mBio.02839-20-st002.pdf]

Hit Compound	PubChem CID	CAS Number	RTECS Number	Route	LD_{50} (mg/kg)	References
**Niclosamide**	4477	50-65-7	VN8400000	Oral	1000	*[1], [2]*
				IP	7.5	*[1], [3]*
**Pyrimethamine**	4993	58-14-0	UV8140000	Oral	92	*[4], [5]*
				IP	74	*[4], [6]*
**Vandetanib**	3081361	443913-73-3	VA0918650	Oral	>1000	*[7]*

Table S2. Acute toxicity of computationally identified, FDA-approved hit compounds. Toxicity for hits identified in the SWEETLEAD database as having chemical similarity to query molecules as reported in the PubChem database. Data for oral and intraperitoneal administration are reported, where available. LD_{50} values represent acute toxicity, for a single dose by the relevant delivery route.

References

1. *National Center for Biotechnology Information. PubChem Database. Niclosamide, CID=4477, https://pubchem.ncbi.nlm.nih.gov/compound/Niclosamide#section=Acute-Effects (accessed on June 23, 2020)*
2. *Wirksubstanzen der Pflanzenschutz und Schadlingsbekampfungsmittel, Perkow, W., Berlin, Verlag Paul Parey, 1971-1976.*
3. *Arzneimittel-Forschung. Drug Research., 10(884), 1960 [PMID:13712649].*
4. *National Center for Biotechnology Information. PubChem Database. Pyrimethamine, CID=4993, https://pubchem.ncbi.nlm.nih.gov/compound/Pyrimethamine#section=Acute-Effects (accessed on June 23, 2020)*
5. *British Journal of Pharmacology and Chemotherapy., 6(185), 1951 [PMID:14848451]*
6. *European Journal of Medicinal Chemistry--Chimie Therapeutique., 9(658), 1974*
7. *National Center for Biotechnology Information. PubChem Database. Vandetanib, CID=3081361, https://pubchem.ncbi.nlm.nih.gov/compound/Vandetanib#section=Toxicity-Summary (accessed on June 23, 2020)*
