# Supplementary material for: A Targeted Computational Screen of the SWEETLEAD Database Reveals FDA-Approved Compounds with Anti-Dengue Viral Activity
Source: mBio. 2020 Nov 10;11(6):e02839-20. doi: 10.1128/mBio.02839-20 (PMC7667029; doi:10.1128/mBio.02839-20)
Supplement: FIG S2 [file mBio.02839-20-sf002.pdf]

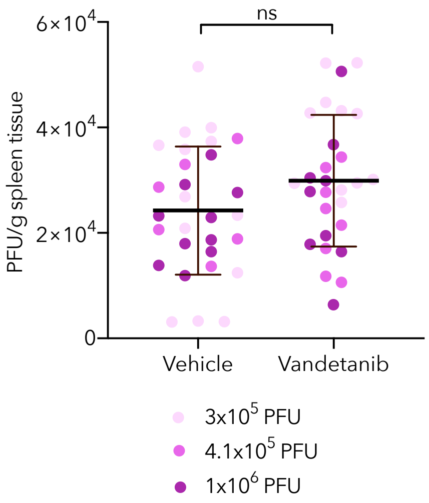


**Fig S2: Vandetanib treatment in dengue-infected mice did not affect splenic titers.** AGB6 mice were infected with $3x10^5$ (light pink), $4.1x10^5$ (magenta), or $1x10^6$ (purple) PFU dengue virus. Mice were treated twice daily with 15 mg/kg (30 mg/kg per day) vandetanib or drug vehicle alone, beginning 4 h prior to infection. Splenic titer at 4 days post-infection is shown. Data were analyzed by two-tailed, unpaired t-test.
