# Supplementary material for: A Targeted Computational Screen of the SWEETLEAD Database Reveals FDA-Approved Compounds with Anti-Dengue Viral Activity
Source: mBio. 2020 Nov 10;11(6):e02839-20. doi: 10.1128/mBio.02839-20 (PMC7667029; doi:10.1128/mBio.02839-20)
Supplement: FIG S3 [file mBio.02839-20-sf003.pdf]

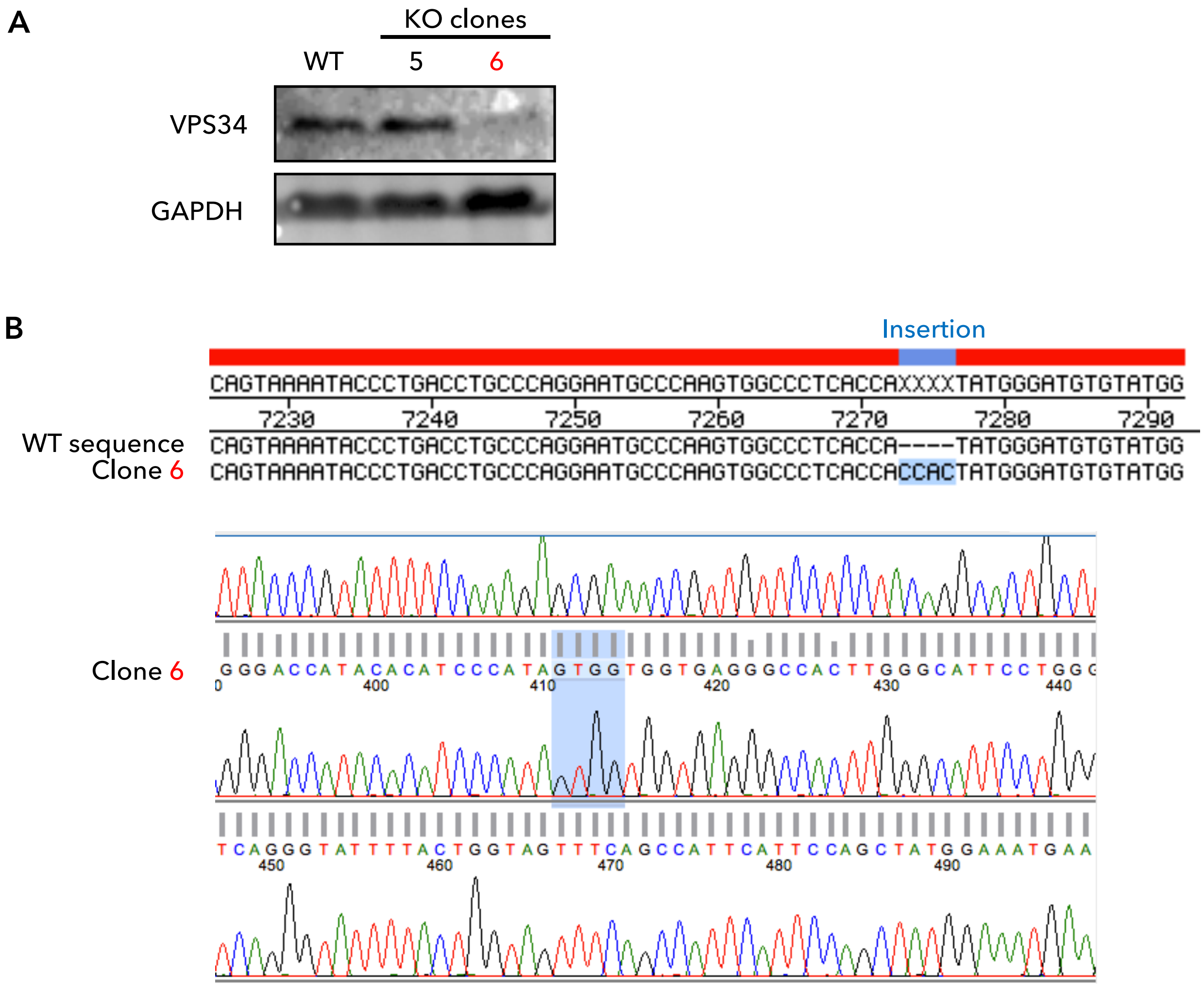

Fig S3. Validation of CRISPR-Cas9 VPS34 knockout (KO) Huh7 cells by immunoblot and Sanger sequencing. VPS34 was knocked out in a Huh7 background through insertion of 4 bp in Exon 3, as described previously *(1)*. (A) Protein lysates from wildtype Huh7 and two separate VPS34 KO clones (clones 5 and 6) were obtained, separated by SDS-PAGE, and immunoblotted using antibodies targeted to VPS34 and GAPDH. Efficient knockout of VPS34 protein expression was observed in clone 6. (B) Genomic DNA from VPS34 clone 6 KO cells was isolated, the VPS34 region was PCR amplified around the targeted cut site, and the PCR product was sequenced by Sanger sequencing to ensure incorporation of the 4 bp insertion (highlighted in blue) by the guide RNAs. VPS34 knockout of clone 6 was determined to be successful and this clone was utilized in further experiments.

References

1. *Abernathy E, Mateo R, Majzoub K, van Buuren N, Bird SW, Carette JE, et al. (2019) Differential and convergent utilization of autophagy components by positive-strand RNA viruses. PLoS Biol 17(1): e2006926. https://doi.org/10.1371/journal.pbio.2006926*
